# The Effectiveness of the Bacteria Derived Extremolyte Ectoine for the Treatment of Allergic Rhinitis

**DOI:** 10.1155/2021/5562623

**Published:** 2021-06-01

**Authors:** Andreas Bilstein, Nina Werkhäuser, Anna Rybachuk, Ralph Mösges

**Affiliations:** ^1^Ursatec GmbH, Marpinger Weg 4, 66636 Tholey, Germany; ^2^bitop AG, Carlo-Schmid-Allee 5, Dortmund, Germany; ^3^Bogomolets National Medical University, Department of Oral and Maxillofacial Surgery, Tarasa Shevchenko Blvd, 13, Kiev, Ukraine 01601; ^4^State Institution “O.S. Kolomiychenko Institute of Otolaryngology of the National Academy of Medical Sciences of Ukraine”, Zoolohichna St, 3, Kiev, Ukraine 03057; ^5^CRI Ltd, Genter Str. 7, 50672 Cologne, Germany; ^6^Institute of Medical Statistics and Computational Biology, Faculty of Medicine, University of Cologne, Kerpener Str. 62, 50937 Cologne, Germany

## Abstract

Nonpharmacological therapies with a good tolerability and safety profile are of interest to many patients with allergic rhinitis, as a relevant proportion of them have reservations about guideline-concordant pharmacological therapies due to their local irritations and side effects. Ectoine is a bacterial-derived extremolyte with an ability to protect proteins and biological membranes against damage caused by extreme conditions of salinity, drought, irradiation, pH, and temperature. Evidence from preclinical and clinical studies attests its effectiveness in the treatment of several inflammatory diseases, including allergic rhinitis. In this review, we analyzed 14 recent clinical trials investigating ectoine nasal spray in patients with allergic rhinitis and/or conjunctivitis, including sensitive patient groups like children or pregnant women. Some studies investigated monotherapy with ectoine; others investigated combination therapy of ectoine and an antihistamine or a corticosteroid. Analysis of the study results demonstrated that patients with mild-to-moderate symptoms of allergic rhinitis can be successfully treated with ectoine-containing nasal spray. When applied as monotherapy, ectoine exerted noninferior effects compared to first-line therapies such as antihistamines and cromoglicic acid. Using ectoine as an add-on therapy to antihistamines or intranasal glucocorticosteroids accelerated symptom relief by days and improved the level of symptom relief. Importantly, concomitant treatment with ectoine was proven beneficial in a group of difficult-to-treat patients suffering from moderate-to-severe rhinitis symptoms. Taken together, the natural substance ectoine represents a viable alternative for allergic rhinitis and conjunctivitis patients who wish to avoid local reactions and side effects associated with pharmacological therapies.

## 1. Introduction

Ectoine is a natural extremolyte found in bacteria which grows under extreme conditions of salinity, drought, irradiation, pH, and temperature [1, 2]. Ectoine binds strongly to water molecules [3], thereby forming a protective hydrate shield around proteins and other biomolecules [4]. It works via a mechanism known as “preferential exclusion” [5]; i.e., it is preferentially excluded from the hydrate shield, leading to the alteration of the aqueous solvent structure [6, 7], which protects proteins from damage and irreversible denaturation and stabilizes biological membranes [3, 8–10]. In preclinical studies, ectoine was shown to protect lung and skin cells against the damage induced by toxic pollution particles and to prevent the subsequent activation of inflammatory cascades [11–16]. A similar effect was observed in model systems for inflammatory bowel disease [17]. Promising findings from clinical trials harnessed ectoine as a therapeutic agent for several inflammatory diseases such as atopic dermatitis [18], upper airway inflammations like pharyngitis/laryngitis [19, 20], rhinosinusitis and acute bronchitis [21] as reviewed by Casale and colleagues [22], rhinitis sicca [23], chemotherapy-induced mucositis [24], and also lung inflammation caused by environmental pollutants [25], even in long-term applications in children to prevent upper respiratory infections [26] or treatment of vernal keratoconjunctivitis [27].

The global prevalence of all allergic diseases is reported to be 20-30% [28], resulting in a high pressure on the social economic systems. The Global Allergy and Asthma European Network report indicated that cost savings of over EUR 100 billion could be realistically expected through better treatment of allergic diseases [29]. The 2008 and 2020 Allergic Rhinitis and its Impact on Asthma (ARIA) guideline provides physicians with a treatment algorithm for allergic rhinitis and conjunctivitis depending on the severity and duration of symptoms [30, 31]. Pharmacological therapies with oral/topical antihistamines, intranasal glucocorticosteroids (INCS), oral glucocorticosteroids, decongestants, leukotriene receptor antagonists, and cromones are considered the mainstay of allergic rhinitis treatment. However, despite the myriad of treatment options with pharmacological drugs, a relevant proportion of patients with moderate-to-severe symptoms are still not sufficiently treated [32–36]. For instance, a study reported that about 60% of allergic rhinitis sufferers in the U.S. are “very interested” in trying out new medications [37]. Furthermore, some patients are reluctant to use pharmacological therapies for fear of local irritations and side effects associated with sedative antihistamines as well as tachyphylaxis under long-term use of nasal decongestants, which can lead to poor medication compliance [38, 39]. Therefore, nonpharmacological therapies with an advantageous tolerability and safety profile are of interest to many patients with allergic rhinitis and conjunctivitis.

For treatment of allergic rhinoconjunctivitis, ectoine nasal sprays and eye drops are already on the market as medical devices in several countries. Eichel and colleagues have published a meta-analysis on selected clinical studies recently [40]. Since the ectoine nasal spray was the first ectoine-containing product envisioned as a nonpharmacological therapeutic agent for allergic rhinitis, a considerable number of clinical trials with this product have meanwhile been performed. Following the initial controlled trials comparing ectoine to standard pharmacological therapies, several real-life, interventional, or noninterventional trials have been conducted. In this article, we systematically reviewed the literature on treatment of allergic rhinitis with ectoine-based nasal sprays to disseminate the most current evidence for the treatment of allergic rhinitis with this interesting substance.

## 2. Methods

This review was conducted in accordance with the Preferred Reporting Items for Systematic Reviews and Meta-Analyses (PRISMA) statement [41]. In order to evaluate the quality of the selected studies, a Jadad score was allocated for each trial to assess methodological quality [42] as indicated in Table 1.

### 2.1. Objectives and Search Strategy

Initial search databases were PubMed, Google Scholar, and Ovid; search language was English. After the search in Google Scholar and PubMed reported several articles in Russian and Ukrainian language, we extended the search to Elibrary.ru and to the National Library of Ukraine and included Russian/Ukrainian language as search criterion. The country of origin and languages were not limited; the period was set to the beginning of 2010 to 15-Nov-2020.

**Figure 1:**
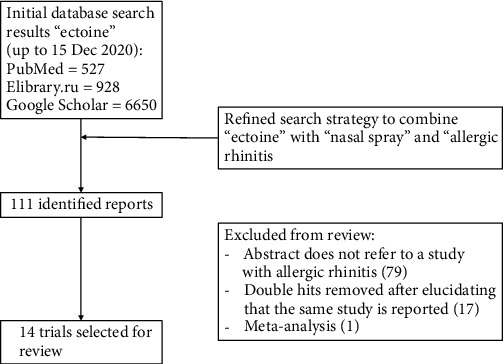
PRISMA diagram of the systematic search performed.

The following key word/medical subject headings were used as search terms: “ectoine” and “nasal spray”, “ectoine” and “allergic rhinitis”, “ectoine” and “nasal irritation”, “ectoine” and “allergic rhinoconjunctivitis”, “ectoine” and “allergy”, “ectoine” and “hay fever”, “ectoine” and “nose”. The search was limited to clinical trials describing the application of ectoine nasal spray in allergic rhinitis, independent on the design of the study (including controlled, noncontrolled, interventional, and noninterventional studies). Only studies published in peer-reviewed journals or presented on scientific congresses were considered. Reference lists of the selected articles were assessed, and additional references fitting the subject of this review were included. Reviews, systematic reviews, meta-analysis, case series, publications containing preclinical data, letters, editorials, errata, and reports of pooled data were excluded (Figure 1).

### 2.2. Search Results

Following the search strategy described above, a total of 14 relevant human studies performed between 2010 and 2019 investigating ectoine nasal spray (ENS) in treatment of allergic rhinitis were selected and further evaluated.

### 2.3. Study Design and Study Population

Except for one trial [43], all trials were real-life studies applying the ectoine product (s): a preservative-free nasal spray containing 2% ectoine, 0.9% sea salt, and water in the 3K System and, where applicable, preservative-free eye drops containing 2% ectoine, 0.35% hydroxyethyl cellulose, 0.35% sodium chloride, citrate buffer, and water, according to the instruction for use over a rather short period of time (1 week to 4 weeks, depending on trial) either as monotherapy or in combination with other interventions. All studies were performed with patients (adults and children) manifesting clinical symptoms characteristic of allergic rhinitis, which had been diagnosed by radioallergosorbent or skin prick test.

Patient-reported symptoms (diary) were used as a primary outcome parameter in all studies. These scoring differed greatly and ranged from combined visual analogue scales for all symptoms, to individual scales for up to 8 symptoms. Consequently, summary scores were calculated differently. Several trials applied additional methods, such as rhinocytogram, rhinoscopy, or eosinophil counting [44, 45].

In all selected studies, a saline-based nasal spray with 2% ectoine was applied. Comparator products are listed as follows:

Intranasal corticosteroids (INS) (mometasone, fluticasone, beclomethasone) [46–48]

Oral or local antihistamine or local cromoglicate [49, 50]

Standard of care (guideline conform use of antihistamine and cromoglicate) [51–53]

Intranasal isotonic salt solution [43, 54, 55]

Only 3 of the selected studies applied a randomization [43, 46, 50], whereas 3 did not carry out randomization due to local regulatory restrictions [47, 49]. Two studies were single-arm trials [44, 45], and the remaining studies did not present any information regarding randomization.

From the 14 studies, 2 have been single-armed [44, 45], and one triple-armed [46]. The 11 other studies were 2-armed. Of those 11 studies, 2 studies applied a crossover design [43, 49], and two studied 2 arms, but not comparative [48, 54]. The other 7 studies applied a comparative design including various comparators [47, 49–53, 55].

A total of 681 subjects were studied in the 14 selected trials. Overall, out of the 462 patients applying ENS, 319 patients used ENS alone, and 171 in total used ENS combined with other pharmacotherapies. 315 patients used a study-specific comparator. Six clinical trials specifically studied the effect in children and adolescents (286/681), with the youngest child being 3 years old. All trials except one included both male and female patients (excluding pregnant women). Ryabova et al. carried out a study on pregnant women (45/681).

Three studies were conducted in Germany, one in Canada, one in Ukraine, one in Kazakhstan, and the remaining 8 in Russia.

Additional details are listed in Table 1.

## 3. Results from the Reviewed Clinical Trials

### 3.1. Safety of Ectoine Nasal Spray

All studies evaluated the safety of ENS, covering also the very sensitive patient groups of children and pregnant women. None of the studies reported a serious adverse effect. Among the very low rate of reported adverse effects which have been reported in total, no irreversible AE has been documented. All authors attribute an excellent safety profile towards the ENS (Table 1).

### 3.2. Efficacy and Effectiveness of Ectoine Nasal Spray

The 14 studies analyzed can be categorized into three main groups:

- Application of ENS concomitantly with drugs compared to application of drugs alone [46, 50–53](Table 2)
- Application of ENS alone compared to other therapies or placebo [43, 47, 49, 55] (Table 3)
- Application of ENS only without comparator [44, 45, 48, 54] (Table 4)

## 4. Discussion

### 4.1. Study Design

In this systematic review, several studies reporting on the effect of ectoine nasal spray in allergic rhinitis were analyzed. Although many of the studies were not published internationally (especially the Russian/Ukrainian studies), the studies were comparable regarding the studied indication (allergic rhinitis) and the primary outcome parameter (patient-reported symptoms). However, they differed in terms of efficacy readout, study population (children, adults, pregnant women), study duration (1 to 8 weeks), and also design (comparative studies, add-on studies, noncomparative studies, studies with parallel treatments of the eyes and nose). Only one trial was placebo controlled, and the overall number of patients per trial with medium 48 patients was rather small. Furthermore, many study details were missing, e.g., information whether the trial was blinded or randomized (e.g., Kayb et al. [51]), or only limited information was given regarding the presence of adverse effects in most trials. A CONSORT description was also missing in 9 of 14 studies, which is in line with the fact that most of the studies were not randomized clinical trials. These limitations influence the quality of the studies, resulting in an average Jadad score of 1-2 points (see Table 1).

The differences in study designs impede to cluster and meta-analyze the data. Nevertheless, the 14 selected trials show a clear picture on the potential efficacy/effectiveness and safety of ectoine nasal spray in the treatment of allergic rhinitis.

### 4.2. Monotherapy with Ectoine

Results from real-life studies constitute an important element of evidence-based medicine since they reflect the effectiveness of the treatment with all the confounding factors as per routine medical practice. Eichel et al. [40] conducted a meta-analysis of four clinical trials comparing ectoine to azelastine [49], cromoglicic acid [49], beclomethasone [47], and placebo. These studies were also included in the review presented here. Results from the meta-analysis by Eichel and colleagues show that, after seven days of treatment with ectoine nasal spray, both nasal and ocular symptoms were significantly alleviated and an especially marked improvement was observed in the symptom of nasal obstruction. The authors concluded that the effectiveness of ectoine was noninferior to that of standard “over the counter” treatment regimens. These results are in line with those from the other studies reviewed here. Salapatek et al. [43] proved in their placebo-controlled trial that hallmark symptoms of AR can be significantly improved by monotherapy with ENS.

Results of the study conducted by Abdulkerimov et al. [46] demonstrated that treatment with ENS alone improved nasal symptoms significantly, but it was less effective than treatment with INS alone. Likewise, results from the study conducted by Sonnemann et al. [47] confirmed that ENS is less effective than beclomethasone nasal spray. Given that INS are the most efficacious pharmacological treatment for allergic rhinoconjunctivitis [30], it is not surprising that the effectiveness of ENS alone, which still showed an impressive >50% symptom improvement, does not match up to that of INS.

Mokronosova et al. showed in 2 studies that treatment for 14 days with ENS resulted in successful treatment of 58.8% and >90% of patients, respectively [44, 45]. According to Abdulkerimov et al. [46], significant nasal symptom relief was evident within 18 to 21 days in moderate-to-severe rhinitis patients who underwent treatment with ENS. According to Sonnemann et al., ENS reduced the nasal symptoms of mild-moderate patients already significantly within the first day of treatment [47]. Furthermore, it has been shown in other trials that patients with severe rhinitis symptoms are difficult to treat. Even with the most effective intranasal formulation, combined azelastine and fluticasone furoate, patients with moderate-to-severe allergic rhinitis showed a relatively low responder rate of 12.4% [56] or 16.7% [57] after 14 days of treatment. In general, only 30.3% of grass pollen-allergic patients and 54.3% of those suffering from birch pollen allergy attain symptom control with guideline-concordant pharmacotherapy [58]. All studies investigating a monotherapy with ectoine invariably attested positive effects of ectoine monotherapy in alleviating symptoms of allergic rhinitis. Taking the baseline symptom scores into consideration, these results permit the conclusion that patients with mild-to-moderate symptoms could be successfully treated with ectoine alone; however, monotherapy with ectoine should not be considered in patients with severe symptoms. In head-to-head comparison studies, ectoine was proven superior to isotonic (sea) salt solutions [43], equivalent to antihistamines (azelastine) and cromoglicic acid [49] but less effective than INS (beclomethasone, mometasone, fluticasone) [46, 47].

### 4.3. Combination Therapy with Ectoine

In accordance with various guidelines, combination therapy is commonly used to treat allergic rhinitis. A large-scale, real-world survey on the prescribing behavior of UK physicians showed that 20-40% of patients who used monotherapy with antihistamines at the beginning of the pollen season and 25-50% of those who used INS used add-on therapy during the pollen season [36]. In patient-based surveys, the percentage of patients who used both, prescription and nonprescription products, was higher (53.0-70.4%), because patients commonly purchase symptomatic medication for allergic rhinitis over the counter in addition to the prescribed drugs [59–61]. These figures warrant the search for an effective treatment combination for patients who suffer from rhinitis symptoms despite the use of first-line therapy.

Evidence supports the use of combination therapy in allergic rhinitis, specifically combinations of pharmacological drugs. The combination of oxymetazoline and mometasone furoate nasal spray showed greater reductions in allergic rhinitis symptoms than mometasone furoate nasal spray alone [62]. Likewise, the combination of oxymetazoline and fluticasone furoate was also superior to both monotherapies [63]. Greiwe and Bernstein [64] conducted a systemic review of combination pharmacotherapy for rhinitis: they concluded that two combinations—intranasal antihistamine (azelastine) with INS and INS with nasal decongestants—are advantageous for patients with complex rhinitis symptoms in terms of symptom control and a preponderance of benefit over harm. The ARIA guidelines 2016 revision recommends the combination of intranasal/oral antihistamines and INS for patients with seasonal allergic rhinitis; the combination of INS and intranasal antihistamines acts faster than INCS alone and thus might be preferred by patients [31].

We reviewed five studies investigating ENS (nonpharmacotherapy) as add-on to pharmacotherapy (antihistamine, cromoglicate, and/or INS), in which monotherapy using either ectoine or pharmacotherapy was used as a comparator. Regardless of treatment regimens, combination therapy with ENS consistently elicited not only greater but also faster symptom relief than did antihistamine alone and INS alone [46, 50, 51]. In the study by Minaeva and Shiryaeva [50], treatment with oral antihistamine alone showed only modest effects in children and adolescents with mild-to-moderate symptoms of allergic rhinitis, whereas those applying ENS additionally were mostly “cured” after treatment end. According to Abdulkerimov et al. [46] and Bardenikova et al. [52], ENS improved the effectiveness of INS. The study of Abdulkerimov et al. was of particular interest to us as the combination of ectoine and INS showed the best treatment effect in difficult-to-treat patients with moderate-to-severe rhinitis symptoms. This trend was also observed for the most effective intranasal formulation (azelastine and fluticasone furoate) in patients with moderate-to-severe rhinitis who exhibited complete or near-complete symptom relief faster than those receiving either fluticasone furoate or azelastine alone [46].

Compared to the combinations of antihistamines with INS or nasal decongestants with INS, the advantages of the combination with ENS lie in its excellent tolerability and safety profile, given that most pharmacological drugs are associated with considerable local irritations and side effects [65, 66]. For instance, it is well known that oxymetazoline might trigger rhinitis medicamentosa, and intranasal steroids might cause stunted growth in children [65, 67]. The combination of fluticasone furoate and azelastine is not indicated for patients under 12 years old because of lack of corresponding data, but the combination of ectoine and antihistamine is suitable for children, as shown by different studies [50–53].

Taken together, the increased effectiveness and time advantage observed in the combination therapy with ectoine were consistent across all studies described above. Thus, ectoine can be deemed a safe and effective add-on to guideline-concordant therapy with antihistamines, cromoglicic acid, or INS.

### 4.4. Concomitant Use of Ectoine Eye Drops

In two of the selected studies [43, 49], ectoine-containing eye drops were applied together with the ENS in order to treat ocular symptoms (allergic rhinoconjunctivitis). Results showed significant and clinically relevant improvement of allergic ocular symptoms such as watery eyes and itching. Although this review concentrates on allergic rhinitis and ectoine nasal spray, it is worth to mention that these results are in line with other studies showing positive effects of ectoine-based eye drops for the treatment of allergic conjunctivitis [68–71].

### 4.5. Treatment of Sensitive Patient Groups

A total of seven studies examined the effects of ectoine nasal spray in the very sensitive patient groups of children and adolescents (6 studies, [48, 50–53, 55]) and pregnant women (1 trial, [54]). The results show that ENS shows efficacy/effectiveness in these sensitive patient groups and combines this with its excellent safety profile of a nonpharmacological treatment.

## 5. Conclusions

In this review, we provide evidence based on the review of 14 independent studies from 4 countries that patients with mild-to-moderate symptoms of allergic rhinitis can be successfully treated with ectoine-containing nasal spray. ENS alone exerts noninferior effects compared to first-line therapy such as antihistamines and cromoglicic acid. Using ENS as an add-on therapy to antihistamines or INS accelerated symptom relief by up to 7 days. This combination strategy was proven to be beneficial in a group of difficult-to-treat patients suffering from moderate-to-severe rhinitis symptoms.

This review of 14 studies extends our knowledge about the substance ectoine and their potential applicability in the treatment of allergic rhinitis by providing mainly patient-reported outcomes in real-world settings under different regional settings with different allergen exposure, standard of care, and different patient groups including very sensitive patient groups. Especially, the combination of different treatment approaches like ectoine treatment in combination with other medications (such as antihistamines or INS) showed additional potential for increased efficacy in patients with allergic rhinitis.

Although the studies have their limitations in design, patient number, and reporting, the following final conclusion can be made: ectoine is a natural substance with an excellent tolerability and safety profile and thus is maybe a viable alternative for allergic rhinitis patients who wish to avoid local reactions and side effects associated with pharmacological therapy. Larger scale controlled and randomized studies would be desirable to further verify the obtained results.

## Figures and Tables

**Table 1:** Details on the selected studies. ACT: asthma control test; AE: adverse event; AR: allergic rhinitis; ASIT: allergen-specific immunotherapy; IAR: intermittent allergic rhinitis; INS: intranasal steroids; SAE: serious adverse event; TNNSS: total nonnasal symptom score; TNSS: total nasal symptom score; TOSS: total ocular symptom score.

Authors, year, study location [reference]	Indication of study	Comparator	Study design	*N* of patients (total and by groups)	Patient population, age (years) of participants	Inclusion criteria/exclusion criteria (only AR-specific criteria listed)	Treatment duration, dosage, and number of visits	Outcome parameters	Reported side effects	Jadad scale (criteria)
Minaeva and Shiryaeva, 2015, Russia [50]	Allergic rhinitis	Oral antihistamine	R, C, OL	Total: 50 Ectoine + antihistamine: 30 antihistamine alone: 20	Children & adolescents Age range: 3-17 Mean age: 10.6	Inclusion: diagnosis of seasonal AR, sensitization to tree pollen, received an antihistamine product Exclusion: taking INS	21-day (3 weeks) treatment Ectoine: 3-4 times daily Antihistamine: age-appropriate dose 3 visits	Nasal congestion, rhinorrhea, itching, sneezing	No AE reported	3 (A, B, E)
Abdulkerimov et al., 2016, Russia [46]	Allergic rhinitis	Intranasal glucocorticoid	R, C, OL	Total: 90 Ectoine alone: 30 INS alone: 30 Ectoine + INS: 30	Adults Age range: 18-65 Mean age: not assessed	Inclusion: diagnosed intermittent AR, during a period of exacerbation of symptoms, admissible accompanying pharmacotherapy Exclusion: pregnant and nursing women, intolerance to one of the substances, usage of other antiallergy agents, nose surgery within 6 weeks before the study, signs of bacterial disease, flu, antibacterial treatment, stomatic disease	60-day treatment Ectoine: 1-2 sprays 3 times a day INS: 2 doses once a day 4 visits	Sneezing, itching, rhinorrhea, nasal congestion, dry mucosa, cough, fatigue, eosinophils, unpleasant smell in the rhinopharynx, rhinoscopy status, video endoscopic investigation status of the nasal cavity, cytological investigation status of the nasal secretion	No AE reported for the ectoine treatment, 4 patients from the INS-alone group reported increased dryness of the nasal mucosa	2 (A, B)
Kayb et al., 2016, Russia [51]	Allergic rhinitis	Anti-inflammatory therapy (standard of care)	C, OL	Total: 60 Ectoine + anti − inflammatory therapy: 30 Anti-inflammatory therapy alone: 30	Children & adolescents Age range: 5-17 Mean age: not assessed	Inclusion: diagnosed seasonal allergic rhinitis, Exclusion: AR flare-ups associated with an acute respiratory disease with increased body temperature, as well as children with an AR flare-up, atopic dermatitis, and bronchial asthma	28-day treatment (4 weeks) Ectoine: as prescribed Anti-inflammatory: as prescribed 4 visits	Rhinorrhea, paroxysmal sneezing, nasal congestion clinical manifestations of the flare-ups and the severity of AR in the course	No AE reported	1 (E)
Bardenikova et al., 2016, Russia [52]	Allergic rhinitis	INS	OL, C	Total: 30 Ectoine: 17 Ectoine + INS: 13	Children & adolescents Age range: 7-17 Mean age: 12.4	Inclusion: diagnosis of all year-round AR (mild and moderate severity) with clinically significant sensitization to house allergens. The patients were enrolled while experiencing exacerbation of persistent AR, with a total score for nasal symptoms (TNSS) of 2 points or more Exclusion: seasonal exacerbations caused by associated pollinosis	21-day treatment Ectoine: 2 doses per nostril, 3 times per day INS: as prescribed 2 visits	TNSS, rhinoscopy status, rhinocytogram status, tolerability, compliance, adverse effects, blood test, cytomorphology (cell count), pediatric ACT, eosinophilic cytosis	3 AEs were reported. 2 increased sneezing immediately following application of the spray; 1 short nasal bleeding during the administration	1 (E)
Skosarev et al., 2015, Kazakhstan [53]	Allergic rhinitis	Standard of care	OL, C	Total: 36 Ectoine: 18 Standard of care: 18	Children & adolescents Age range: not assessed Mean age: not assessed	Inclusion: children with diagnosis of AR	10-day treatment Ectoine: as prescribed Standard of care: as prescribed	Itchiness, periodic sneezing, coughing, voice changes, night snoring, emotional profiles	No information given	0
Salapatek et al., 2011, Canada [43] (manuscript accepted for publication)	Allergic rhinoconjunctivitis	Placebo	R, C	Total: 46 Ectoine: 46 Placebo: 46	Adults Age range: 18-65 Mean age: 43.9 ± 11.3	Inclusion: history of seasonal AR, positive skin prick test, TNSS ≥ 6/2, TOSS ≥ 4/9 Exclusion: usage of antiallergic medication before study start and throughout the study	14-day treatment per treatment type crossover after 7 days washout Ectoine: 1 spray/nostril 3 times per day Placebo: 1 spray/nostril 3 times per day 5 visits	Primary: TNSS (including sneezing, itchy nose, runny nose and nasal congestion) and TOSS (including watery eye, itchy eye, red eye) Secondary: TNNSS (including watery eye, itchy eye, red eye, and itchy ear/palate), and acoustic rhinometry measurements	6 AEs reported during ectoine treatment. During placebo treatment, 5 AEs were reported. No SAEs occurred	5 (A-E)
Sonnemann et al., 2014, Germany [47]	Allergic rhinitis	Beclomethasone	OL, NI, C	Total: 50 Ectoine: 25 Beclomethasone: 25	Adults Age range: 18-65 Mean age: 33.3	Inclusion: 18-70 years, diagnosed seasonal allergic rhinitis, TNSS > 6 Exclusion: not described	14-day treatment Ectoine: 3 times daily, Beclomethasone (0.05 mg): 2 times daily, 2 visits	Primary: nasal obstruction, rhinorrhea, nasal itching, sneezing Secondary: itchy ear/palate, efficacy, tolerability	3 AEs, 2 in the ectoine group (not related), 1 in the beclomethasone group (probably related)	1 (E)
Werkhäuser et al., 2014, Germany [49]	Allergic rhinitis	Azelastine	OL, NI, C	Total: 48 Ectoine: 22 Azelastine: 26	Adults Age range:18-65 Mean age: 35	Inclusion: patients aged 18-70, proven allergy in prick test, acute symptoms in nose and eyes Exclusion: pregnant and nursing women, drug addicts, patients with intolerance against ingredients of treatments, previous eye or nose surgery	7-day treatment Ectoine: one eye drop per eye and one puff of the nasal spray per nostril four times per day Azelastine: One eye drop, one puff nasal spray, both twice per day 2 visits	Nasal obstruction, rhinorrhea, sneezing nasal itching, conjunctivitis, eye itching, tearing, palate itching	8 AEs: 2 cases of burning of eyes and itching of the throat in the ectoine group, and 6 (4 cases of burning of eyes, 1 case of nausea, and 1 case of headache) in the azelastine group	1 (E)
									No SAE occurred	
Werkhäuser et al., 2014, Germany [49]	Allergic rhinitis	Cromoglicic acid	OL, NI, C	Total: 50 Ectoine: 25 Cromoglicic acid: 25	Adults Age range: 18-65 Mean age: 35	Inclusion: patients aged 18-70, proven allergy in prick test, acute symptoms in the nose and eyes Exclusion: pregnant and nursing women, drug addicts, patients with intolerance against ingredients of treatments, previous eye or nose surgery	14-day treatment crossover after 7 days Ectoine: 5 times per day, cromoglicic acid (20 mg/ml): nasal spray 4 times per day 3 visits	Nasal obstruction, rhinorrhea, sneezing nasal itching, conjunctivitis, eye itching, tearing, palate itching	During the study, no SAE occurred	1 (E)
									No AEs were observed for ectoine containing nasal spray; 15 AEs were recorded for the cromoglicic acid nasal spray	
Kryuchko et al., 2014, Ukraine [55]	Allergic rhinitis	Sea salt solution	OL, NI, C	Total: 60 Ectoine: 38 Sea salt solution: 22	Children & adolescents Age range: 6-14 Mean age: not assessed	Inclusion: sensitized to pollen allergens, sufficient compliance, patients applying antihistamines on demand Exclusion: not described	14-day treatment Ectoine: as prescribed Control: as prescribed 5 visits	Activity, sleep, nasal symptoms, ocular symptoms, emotions	No adverse effects reported	0
Ryabova et al., 2019, Russia [54]	Allergic rhinitis	None	OL	Total: 45 Group persistent allergic rhinitis: 25 Group intermittent allergic rhinitis: 20	Pregnant women Age range: 18-40 Mean age: 30.7 ± 0.7	Inclusion: pregnancy, proven case of AR at the time of examination, aged 18 to 40 years, presence of 2 or more typical signs of AR, bright-red mucosa in aggravated seasonal allergic rhinitis, cyanotic or gray mucosa in perennial allergic rhinitis, the mucosa spotting (“marbling”) (Voyachek's symptom) Exclusion: present infectious diseases, history of alcohol or drug abuse, problems that may limit the patient's ability to follow the protocol requirements, participation in another clinical trial during the three months preceding the screening visit, any other conditions that make it difficult to participate in the study	All patients received 1-2 doses of ectoine 3-4 times a day or for 10 days before contact with allergens	Rhinoscopy status, laboratory tests, mucociliary clearance	None reported	1 (E)
Kamaev and Трусоbа, 2015, Russia [48]	Allergic rhinitis	None	OL	Total: 50 Ectoine + antihistamine: 22 Ectoine + INS: 28	Children & adolescents Age range: not assessed Mean age: not assessed	Inclusion: diagnosis of persistent AR, domestic or epidermal sensitization, exacerbation of the disease at the first visit Exclusion criteria not described	4 weeks of treatment Treatments as prescribed 3 visits	Rhinocytogram status, eosinophil count (laboratory test), result of the anterior rhinoscopy, result of the TNSS	AEs reported during the study were resolved completely by days 8 to 15 of administration	0
Mokronosova et al., 2017, Russia [45]	Allergic rhinitis	None	OL, NI	Total: 34 Ectoine: 34	Adults Age range: 20-45 Mean age: not assessed	Inclusion: confirmed mono allergy to tree pollen, mild-moderate IAR, two courses of ASIT Exclusion: severe IAR, noncompliance, nose surgery, use of antiallergic drugs	Max. 37-day treatment Ectoine: 1-2 sprays 3-4 times daily (or as necessary) 2 visits	Nasal obstruction, sneezing, nasal itching, skin test, laboratory test with specific serum IgE antibodies, cytological analysis of nasal secretions, otorhinolaryngological examination	No AE reported	1 (E)
Mokronosova et al., 2019, Russia [44]	Allergic rhinitis	None	OL, NI	Total: 30 Ectoine: 30	Adults Age range: 18-60 Mean age: not assessed	Inclusion: tree pollen sensitization confirmed positive test for IgE antibodies Exclusion: severe IAR, noncompliance, nose surgery, use of antiallergic drug	7-day treatment Ectoine: 1-2 sprays 3-4 times daily (or as necessary) 2 visits	Nasal obstruction, sneezing, nasal itching, skin test, laboratory test with specific serum IgE antibodies, cytological analysis of nasal secretions, otorhinolaryngological examination	No AE reported	0

Study design: randomized (R); controlled (C); open-label (OL); noninterventional (NI).

**Table 2:** Application of ectoine in combination with drugs compared to treatment with drugs alone. AR: allergic rhinitis; ENS: ectoine nasal spray; GC: glucocorticoid; INS: intranasal steroids; TNSS: total nasal symptom score.

Author	Treatment groups	Patient reported outcome (nasal symptom scores)	Additional treatment effects observed (nonexhaustive)
Minaeva and Shiryaeva, 2015 [50]	Group 1: ENS + oral antihistamine	In group 1, TNSS was reduced from 4.6 ± 0, (day 1) to 0.4 ± 0.1 (day 21). In group 2, TNSS was reduced from 3.9 ± 0.3 (day 1) to 3.5 ± 0.2 (day 21), respectively. The difference between the groups was significant (*p* = 0.003) Significant differences of single symptoms: For nasal congestion on day 14 (*p* = 0.01), rhinorrhea on day 15 (*p* = 0.036), and nasal itching and sneezing on day 17 (*p* = 0.02), all favoring group 1.	Reduction of ocular itching was higher for group 1 on day 18 (*p* = 0.007). Conjunctival hyperemia was significantly more severe in group 1 at baseline and became comparable between the two groups on day 10 and significantly milder in group 1 on day 19 (*p* = 0.015). Additional symptomatic medication for rhinitis was used significantly less in group 1 (2 out of 30 patients), compared to group 2 (10 out of 20 patients) (*p* = 0.002).
	Group 2: oral antihistamine		
Abdulkerimov et al., 2016 [46]	Group 1: ENS	All 3 treatments reduced nasal symptoms (sneezing, itchy nose, discharges, nasal congestion, unpleasant smell, cough, fatigue) significantly (*p* < 0.05), group 2 had the highest effect, followed by group 3, with group 1 having the lowest, but still a significant effect against baseline.	Diminution of symptoms (in days) reflected the results from the symptom score: In group 2, symptoms diminished faster than in group 3, followed by group 1.
	Group 2: ENS + INS		
	Group 3: INS		
Kayb et al., 2016 [51]	Group 1: ENS + standard of care	In group 1, a positive dynamic of disease symptoms was observed in 92.3% (*p* < 0.05) of mild cases after 2 weeks, and in 60% (*p* < 0.05) in group 2. For the moderate form of AR, the efficacy of combined therapy in group 1 was 67% versus 50% in group 2 (*p* < 0.05). Based on patient questionnaires, the symptoms in mild AR were down by day 3 to day 5. In group 2, a positive dynamic in AR symptoms was observed by day 7. After 4 weeks, in both groups, 100% of the mild AR patients were free of symptoms. No significant effect was observed in children with mild or severe AR in both groups.	In group 1, intranasal GCs could be stopped at week 4 of observation in 100% of cases with the mild form of AR and in 81.8% of cases with the moderate form of AR. In group 2, most children with the moderate form of AR (78.6%) continued to take intranasal GCs intermittently. In the severe form of AR in children in the group receiving ENS, the dose of intranasal GCs was able to be reduced, in contrast to group 2.
	Group 2: Standard of care		
Bardenikova et al., 2016 [52]	Group 1: ENS + oral antihistamine	The reduction of AR symptoms was significant for both groups (*р* < 0.001): patients in group 1 had stronger changes, as their TNSS was reduced from 2.56 ± 0.2 to 0.79 ± 0.1 after 3 weeks of treatment versus 2.92 ± 0.2 to 1.0 ± 0.1 in group 2 (*p* > 0.05). Before enrolment, patients in group 2 had more severe AR symptoms (2.92 ± 0.2 points) when compared to group 1 (2.56 ± 0.2, *р* > 0.05), mainly due to nasal congestion severity. An overall trend in the direction of a reduction in TNSS during the observation supports the efficacy of both groups (*p* < 0.001).	In 62% of pediatric patients of group 2, the TNSS drop was more significant (2-3 points). A similar significant TNSS decrease (2-3 points) in group 1 was only demonstrated in 35% of patients. Rhinoscopy showed improved results for both groups after treatment. Analysis of the AR control index showed better values in patients in group 1.
	Group 2: ENS + INS		
Skosarev et al., 2015 [53]	Group 1: ENS + s standard of care	Nasal breathing and mucous secretion were markedly reduced in group 1. Analysis of nasal itchiness, periodic sneezing, coughing, voice changes, and night snoring showed also definite positive trends (*p* < 0.001). As for group 2, positive trends but not statistically significant differences were shown for the occurrence of itchiness, periodic sneezing, coughing, voice changes, and night snoring.	Interestingly, emotional profiles have been evaluated: analysis performed in the study demonstrated that the most important feature in the evaluation of emotional conditions was the anxiety level, which was found in percent in 84.9 ± 4.8, 32.4 ± 3.1, and 7.4 ± 0.86 of patients in group 1 for days 1, 5, and 10 of observation, accordingly. In group 2, the trend was less prominent: 82.3 ± 5.21, 65.4 ± 3.2, and 12.3 ± 1.61 cases, accordingly.
	Group 2: Standard of care		

**Table 3:** Studies comparing ectoine nasal spray against other therapy or placebo. AR: allergic rhinitis; AUC: area under the curve; EEC: environmental exposure chamber; EED: ectoine eye drops; ENS: ectoine nasal spray; GC: glucocorticoid; INS: intranasal steroids; LSMD: least square mean difference; QoL: quality of life; RQLQ: rhinitis quality of life questionnaire; TNNSS: total nonnasal symptom score; TNSS: total nasal symptom score; TOSS: total nasal symptom score.

Author	Study treatment distribution	Treatment effect patient reported outcome (nasal symptom scores)	Interesting treatment effect on other parameters (nonexhaustive)
Salapatek et al., 2011 [43]	Group 1: ENS/EED	Patients in group 1 had a mean 1.54-fold lower TNSS during posttreatment EEC exposures than placebo patients, though the TNSS decreased not only in group 1 but also in group 2 when compared to baseline. The mean AUC TNSS score was 25.02 ± 0.722 at the EEC screening visit, which was significantly reduced to 20.10 ± 1.31 (-19.7%; *p* = 0.0003) in group 1 at the posttreatment EEC visits. In group 2, the drop was by 12.2% to 21.96 ± 1.21. Intergroup comparison showed that the mean change from baseline AUC of TNSS for group 1 was 61.2% greater compared to group 2 (LSMD: -4.92 vs. -3.05). This difference showed clinically meaningful improvement in group 1 in comparison to group 2 but did only approach statistical significance (*p* = 0.065). Treatment in group 1 resulted in significantly greater relief of the symptom “sneezing” (*p* = 0.020).	In both treatment groups, the TOSS and TNNSS after EEC exposure in comparison to baseline was reduced, but in group 1, we have seen an improvement in ARC symptoms: the mean change from baseline AUC of TNNSS was also significantly lower in group 1 compared to group 2. Mean cross-sectional areas of the nasal cavity were reduced to a lesser extent in group 1.
	Group 2: placebo/placebo		
Sonnemann et al., 2014 [47]	Group 1: ENS	According to the patients' assessment, TNSS values decreased clearly in group 1 (*p* = 0.072, decrease by -12.86%) and a significant decrease was observed in group 2 (*p* < 0.001, decrease by 39.69%). In order to study the time of onset of both treatments, TNSS development within the first 12 hours of treatment was analyzed. Both groups showed a significant decrease of TNSS from the first site visit until the first patient assessment at the end of the first day of treatment (*p* < 0.001 for both groups).	After 14 days of treatment, in the investigator's assessment, both groups showed a significant reduction in TNSS levels. Single symptom score and ear/palate itching analysis and QoL questionnaire revealed only significant changes for group 2 for sneezing and brushing the nose. In group 1, mean values of 1.09 ± 0.78 (mean values of entire study period) reflected moderate efficacy assessed by patients and a value of 1.44 ± 1.00 showed similar judgment by the physicians. In group 2, the efficacy was judged as good by patients (1.73 ± 0.94) and as very good by investigators (2.60 ± 0.58).
	Group 2: INS		
Werkhäuser et al., 2014 [49]	Group 1: ENS/EED	The sum of nasal symptom scores showed a significant decrease from visit 1 to visit 2 (as assessed by physicians): sum scores in group 1 decreased from 20.71 ± 3.52 to 8.52 ± 4.74 (*p* < 0.001) and sum scores in group 2 decreased from 21.73 ± 3.34 to 9.32 ± 6.24 (*p* < 0.001). According to the patients' assessment, values decreased by 23.05% in group 1 (*p* = 0.076) and by 33.14% in group 2 (*p* = 0.02). All single symptoms (nasal obstruction, rhinorrhea, sneezing, nasal itching) decreased significantly in both groups.	As for nasal symptoms, a clear decrease of the symptom palate itching was observed from visit 1 to visit 2: *p* = 0.024 for group 1 and *p* = 0.018 for group 2. Values of the patients' documentation did only reach statistical significance in group 2 (*p* < 0.001). The TOSS decreased significantly from visit 1 to visit 2 in both groups (*p* < 0.001 for group 1, *p* = 0.009 for group 2).
	Group 2: azelastine nasal spray and eye drops		
Werkhäuser et al., 2014 [49]	Group 1: ENS	According to the physician's assessment, TNSS scores decreased significantly for both groups both from visit 1 to visit 2 (*p* < 0.001) and from visit 1 to visit 3 (*p* < 0.001). Scores assessed by patients showed that decreases in TNSS from day 1 to day 7 were not significant, whereas significant decreases in TNSS scores from day 1 to day 14 were shown for group 1 (*p* < 0.001) as well as group 2 (*p* < 0.001). Single symptom scores also decreased significantly in both groups.	The development of the sum of TOSS was assessed by the investigator. It could be confirmed that ocular symptoms decreased significantly from visit 1 to visit 2 (*p* < 0.001 for group 1; *p* = 0.008 for group 2) as well as from visit 1 to visit 3 (*p* < 0.001 for group 1; *p* = 0.003 for group 2).
	Group 2: cromoglicate nasal spray		
Kryuchko et al., 2014 [55]	Group 1: ENS + saline solution	At the end of the treatment course, overall improvement was achieved for both groups but was more prominent in group 1 (improvement of the TNSS by 4.6 vs. 4.2; *p* < 0.05).	Applying the RQLQ, 81.5% of patients in group 1 scored the treatment results as “good,” 15.8% scored the treatment results as “fair,” and only 2.7% continued to use topical antihistamine products due to polyvalent sensitization and the persistent course of allergic rhinitis. In group 2, a “good” score was achieved for 58% of patients, “fair” score was achieved for 25%, and 17% of pediatric patients had to continue intranasal therapy due to persistent symptoms.
	Group 2: saline solution		

**Table 4:** Studies applying ectoine nasal spray without comparator. AR: allergic rhinitis; ENS: ectoine nasal spray; ENT: ear-nose-throat; INS: intranasal steroids; TNSS: total nasal symptom score.

Author	Study treatment distribution	Treatment effect patient reported outcome (nasal symptom scores)	Additional other treatment effect (nonexhaustive)
Ryabova et al., 2019 [54]	Group 1: ENS (persistent allergic rhinitis)	After 10 days of treatment, both groups showed a significant reduction in complaints: the total clinical score developed from 4.6 ± 0.7 points to 0.7 ± 0.4 points in group 1 and from 5.3 ± 1.0 points to 0.4 ± 0.2 points in group 2.	The ENT examination showed a decrease in the severity of inflammatory events in the nasal cavity (*p* < 0.05). Cytological evaluation of nasal secretion in both groups revealed an increase in the relative count of eosinophils and the absolute count of leukocytes. This was mainly due to an increase in neutrophils. A significant decrease in the amount of secretory IgE was observed in group 1 and group 2 (*p* < 0.05).
	Group 2: ENS (intermittent allergic rhinitis)		
Kamaev et al., 2015 [48]	Group 1: ENS plus antihistamines and/or cromoglicate	A decrease in the severity of AR symptoms on the TNSS scale in both groups (−3.2 ± 0.4 points in group 1 and −4.5 ± 0.6 points in group 2) was observed.	As a result of combination therapy in both groups, a marked decrease in both clinical and laboratory activities of inflammation was achieved between visits 1 and 3: a decrease in eosinophil count (−0.7 ± 0.4 in group 1 and −0.6 ± 0.5 in group 2); a decrease in the overall assessment score of the rhinocytogram (−1.1 ± 0.6 in group 1 and −0.9 ± 0.5 in group 2); a decrease in the score of AR exacerbations according to the anterior rhinoscopy data (−3.1 ± 0.9 in group 1 and −3.6 ± 1.1 in group 2).
	Group 2: ENS + INS		
Mokronosova et al.,2017 [45]	Group 1: ENS	Most of the patients (20/34) benefited from treatment with ENS. In 6 out of 34 participants, the disease worsened and 8 patients expressed no difference. Thus, there were 2.6 and 3.3 times more patients in whom use of ENS spray led to decreases in symptoms.	—
Mokronosova et al., 2019 [44]	Group 1: ENS	All patients except one showed a decrease in the intensity of all clinical symptoms of AR.	After a week of use of ENS, the range of eosinophils in the rhinocytogram has not changed. However, the average number of eosinophils tended to decrease from 29% ± 9% to 22% ± 10%.

## Data Availability

Data sharing is not applicable to this article as no datasets were generated or analyzed during the current study.

## Abbreviations

ACT: Asthma control test
AE: Adverse event
AR: Allergic rhinitis
ASIT: Allergen specific immunotherapy
ARIA: Allergic Rhinitis and its Impact on Asthma
AUC: Area under the curve
EEC: Environmental exposure chamber
EED: Ectoine eye drops
ENS: Ectoine nasal spray
ENT: Ear-nose-throat
GC: Glucocorticoid
INS: Intranasal glucocorticosteroids
LSMD: Least square mean difference
QoL: Quality of life
RQLQ: Rhinitis quality of life questionnaire
SAE: Serious adverse event
TNSS: Total nasal symptom score
TNNSS: Total nonnasal symptom score
TOSS: Total ocular symptom score.
